# Vitamin D Levels Are Reduced at the Time of Hospital Admission in Sicilian SARS-CoV-2-Positive Patients

**DOI:** 10.3390/ijerph18073491

**Published:** 2021-03-27

**Authors:** Agostino Gaudio, Andrea Ruben Murabito, Antonella Agodi, Arturo Montineri, Pietro Castellino, D.O.CoV Research

**Affiliations:** 1 Department of Clinical and Experimental Medicine, University of Catania, 95123 Catania, Italy; pcastell@unict.it; 2 San Marco Hospital, 95121 Catania, Italy; andrearubenmurabito@gmail.com (A.R.M.); a.montineri@libero.it (A.M.); 3 Department of Medical and Surgical Sciences and Advanced Technologies “GF Ingrassia”, University of Catania, 95123 Catania, Italy; agodia@unict.it

**Keywords:** COVID-19, vitamin D, Sicily, immune system

## Abstract

The coronavirus disease 2019 (COVID-19) pandemic poses a worldwide healthcare challenge that needs an efficient response. Unfortunately, to date there is no highly effective treatment, so a deep understanding of COVID-19 risk factors could be an important step in treating the disease. Vitamin D affects the immune system in many different ways, and other authors already found that COVID-19 patients have low levels of vitamin D. In our retrospective study, we evaluated the vitamin D status at the time of hospital admission in 50 COVID-19 patients in Sicily, which is the southernmost region of Italy, and compared them with 100 control subjects matched for age and sex. Our data showed markedly low levels of vitamin D in patients with a positive polymerase chain reaction (PCR) for severe acute respiratory syndrome coronavirus 2 (SARS-CoV-2), but no association was found with inflammation markers or clinical severity. Vitamin D levels were reduced at the time of hospital admission in Sicilian SARS-CoV-2-positive patients, but it is not clear whether this condition has an impact on the clinical course of COVID-19.

## 1. Introduction

The coronavirus disease 2019 (COVID-19) pandemic burden is still weighing upon the whole world in 2021. To date, there is no approved highly effective treatment for the disease, so a further understanding of risk factors linked to COVID-19 and its mortality could be of paramount importance [1,2,3,4]. Vitamin D is a hormone obtained from skin exposure to ultraviolet B (UVB) radiation in sunlight. Besides skin production, vitamin D can also be obtained from dietary sources. Vitamin D from the skin or diet is then hydroxylated twice: first in the liver and eventually in the kidney, becoming its active form, 1,25-dihydroxyvitamin D, which binds to the vitamin D receptor (VDR), resulting in activation or suppression of certain genes. Vitamin D has a well-known role in calcium and phosphorus homeostasis and bone metabolism, but many observations in the last decades have pointed out that vitamin D also has a role in innate and adaptive immunity [5].

A very hot topic in the debate in the international scientific community is the possible role of vitamin D in the prevention or treatment of COVID-19 [6,7]. For these reasons, in this observational, retrospective study, we investigated the vitamin D status in a cohort of patients from Sicily (Italy), who were SARS-CoV-2-positive.

## 2. Material and Methods

### 2.1. Data Collection

We evaluated retrospectively the repository data of SARS-CoV-2-positive patients who were consecutively admitted to the Infectious Diseases Unit of San Marco Hospital (Catania, Italy) from March to July 2020. From the total (*n* = 90), we considered only those patients with positive SARS-CoV-2 PCR (polymerase chain reaction) whose vitamin D status was available (*n* = 50).

For each patient, we evaluated clinical history, laboratory exams, outcome (dead or alive), length of hospitalization, and type of oxygen therapy or ventilation needed during hospitalization [none, nasal cannula/Venturi mask/high-flow nasal cannula (HFNC), continuous positive airway pressure (cPAP)/non-invasive ventilation (NIV) or invasive ventilation]. For phosphocalcic biochemical parameters, we considered as control group the first 100 SARS-CoV-2-negative patients matched for age and sex, who were consecutively admitted in the same period to the Unit of Internal Medicine, University Policlinic (Catania, Italy). None of the patients or controls were taking vitamin D supplements at the time of the evaluation or in the previous 3 months. The body mass index (BMI) was calculated for all subjects. The protocol was approved by the local ethics committee (Comitato Etico Catania 1, Azienda Ospedaliero-Universitaria Policlinico “G. Rodolico—San Marco” Catania, approval number 283 of 23 December, 2020) and was conducted in accordance with the Declaration of Helsinki.

### 2.2. Biochemical Analyses

We evaluated the following biochemical parameters: calcium, phosphorus, creatinine, alkaline phosphatase (ALP), 25-OH vitamin D2 (25OHD), parathyroid hormone (PTH), interleukin-6 (IL-6), D-dimer, and C-reactive protein (CRP). According to the Institute of Medicine, vitamin D deficiency is defined as a serum 25OHD level of <12 ng/mL (30 nmol/l) and insufficiency as a 25OHD level of 12–20 ng/mL (50 nmol/l). A 25OHD level of >20 ng/mL was accepted as adequate [8]. The determination of all parameters was performed on fasting morning blood samples within 5 days of hospitalization.

### 2.3. Statistical Analysis

Continuous variables are presented as median (range); categorical variables are presented as percentages. D’Agostino–Pearson normality test was used to confirm that variables were well-modelled by a normal distribution. Clinical and laboratory variables were compared using analysis of variance for continuous variables and chi-squared tests for categorical variables. Pearson correlation coefficient was used to assess the relationship between 25OHD levels and other biochemical parameters. Statistical analyses were performed using NCSS 2007 and PASS 2005 software (Gerry Hintze, Kaysville, UT, USA). A two-tailed *p* < 0.05 was considered significant. Bonferroni’s correction was used for multiple comparisons.

## 3. Results

**Figure 1:**
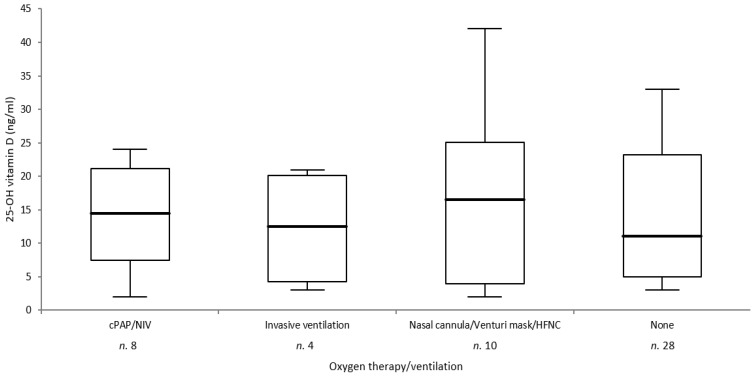
Analysis of variance (ANOVA) displayed as box-plot of serum 25-OH vitamin D levels in patients according to oxygen therapy/ventilatory support (*p* = 0.75). HFNC: high-flow nasal cannula; cPAP: continuous positive airway pressure; NIV: non-invasive ventilation.

**Figure 2:**
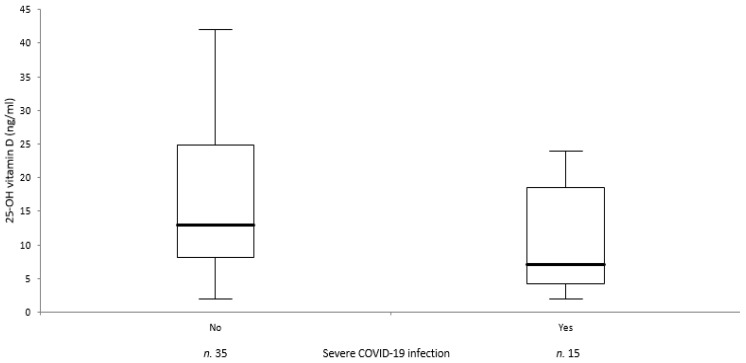
Analysis of variance (ANOVA) displayed as box-plot of serum 25-OH vitamin D levels in patients with severe COVID-19 infection (*p* = 0.08).

In Table 1, we report the clinical and biochemical data of patients and controls included in this study. The two groups were matched for age and sex. There were no significant differences between the two groups for BMI and serum levels of creatinine, ALP, and albumin. Calcium and phosphorous were significantly lower and PTH higher in patients than in controls. Serum levels of IL-6, CRP, and D-dimer were increased in patients with respect to normal ranges (data in controls not available). Median serum levels of 25OHD were significantly lower (12.5 vs. 20.5 ng/mL; *p* < 0.001) in patients than in controls. In particular, 23 patients had values of <12 ng/mL, 11 patients values of 12–20 ng/mL, and only 16 patients values of ≥20 ng/mL. The number of subjects with 25OHD values of <12 ng/mL was significantly higher in patients than in controls. There were no significant differences in 25OHD serum levels between male and female subjects in both groups. Regarding ventilatory support, 28 patients (56.0%) did not need any type of assistance, 10 (20.0%) were treated with oxygen delivered by nasal cannula or Venturi mask, eight (16.0%) were treated with cPAP or NIV, and only four (8.0%) were treated with invasive ventilation. There were no significant differences in 25OHD values as a function of ventilatory support (Figure 1). The 25OHD levels were slightly, but not significantly (*p* = 0.08) reduced in patients affected by severe form of COVID-19 infection (deaths + patients requiring mechanical ventilation or cPAP/NIV; Figure 2). Moreover, we did not observe in patients any correlation between 25OHD values and the length of hospitalization or levels of IL-6, D-dimer or CRP, whereas 25OHD serum levels correlated positively with calcium corrected for albumin (r = 0.405, *p* = 0.003), phosphorus (r = 0.282, *p* = 0.04), and negatively with PTH (r = −0.682, *p* < 0.0001) and age (r = −0.506, *p* = 0.0002). Among the 50 SARS-CoV-2-positive patients that were enrolled, five (10%) died during the period of hospitalization. Also, in this case, we did not observe any significant difference in vitamin D levels. Only two out of five deceased patients and 20 out of 45 surviving patients underwent oxygen therapy or ventilatory support (*p* = 0.77).

## 4. Discussion

To the best of our knowledge, our study is the first conducted in Sicily, the southernmost region of Italy, to evaluate 25OHD levels in patients with positive PCR for SARS-CoV-2. Sicily is characterized by a Mediterranean climate with mild, moderately rainy winters and hot, sunny summers. There are few articles in the literature concerning vitamin D levels in Sicilian subjects, but, in general, the levels reported [9,10] were on overage sufficient according to the Institute of Medicine [8]. In contrast, our patients presented with markedly low levels of vitamin D, with 46% in the range of deficiency.

Hope-Simpson was the first to describe the worldwide seasonality of influenza, and he theorized a ‘seasonal stimulus’ that could explain such behavior [11]. Along with influenza, other viral respiratory tract infections caused by viruses, such as respiratory syncytial virus, parainfluenza viruses, and a large number of common cold viruses, usually peak during wintertime and tend to subside in summer [12,13].

Many observations suggest that vitamin D could be the seasonal stimulus for Hope-Simpson, as fish oil and cod liver oil (oils that contain large amounts of vitamin D) have been found to reduce respiratory tract infections [14]. Moreover, influenza causes many more deaths during cold months when vitamin D levels are the lowest, and respiratory tract infections are more common in children affected with rickets [12].

It was demonstrated that vitamin D has a role in innate and adaptive immunity through many mechanisms. It tends to polarize the adaptive immune system towards responses by T-helper-2 (Th-2) lymphocytes, while suppressing the proliferation of Th-1 lymphocytes. Consequently, the production of some anti-inflammatory cytokines is enhanced, while some pro-inflammatory cytokines are suppressed [15]. Vitamin D also acts as a potent immune regulator by modulating immune-cell activity [16]. In particular, a group of T cells known as regulatory T cells (Tregs) suppresses the immune responses of other T cells in order to prevent excessive autoimmune responses. It was shown that circulating Treg populations are increased by vitamin D administration [17]. Vitamin D also modulates innate immunity through its effects on toll-like receptors (TLRs) that affect, and in the meantime are affected by, VDRs. In addition, it affects the expression of some antimicrobial peptides associated with TLRs that have antiviral effects, such as human beta defensin 2 and cathelicidin [5]. Human cathelicidin peptide LL-37 exerts its antimicrobial effects by recruiting neutrophils, monocytes, and T cells to microbial invasion sites, promoting the clearance of pathogens, inducing apoptosis in infected cells and contributing to innate immunity by transactivating the epidermal growth factor receptor (EGFR) at the epithelial surfaces of airways. Many studies investigating the effects of vitamin D on viral infections featured enveloped viruses. The antiviral effects of cathelicidin, in the form of LL-37, may be partially mediated by envelope disruption, as it mediates the disruption of bacterial membranes [5,18,19,20]. Furthermore, vitamin D has a role in the maintenance of many cellular junctions that act as physical barriers to pathogens [15]. The potential role of vitamin D in respiratory tract infections is also suggested by the fact that cytochrome P450 family 27 subfamily B member 1 (CYP27B1), which converts vitamin D to its active form, is upregulated by viral infections and is highly expressed in lung epithelial cells [16]. Vitamin D deficiency also has a role in other viral infections, as it is related to the replication of the hepatitis B virus (HBV), contributes to inflammation and oxidative stress in patients with hepatitis C virus (HCV), and impairs CD4 T cell recovery in human immunodeficiency virus (HIV)-infected patients treated with highly active antiretroviral therapy (HAART) [16]. Finally, the genetic polymorphism of VDRs is thought to affect the course of several viral infections [16].

To date, there is no certainty about the relationship between low serum vitamin D concentrations and respiratory tract infections because controlled trials investigating such issues had mixed results. [5,21,22,23,24]. One of the foremost pathological mechanisms involved in COVID-19 is thought to be the so-called “cytokine storm”, which is an uncontrolled release of a large number of cytokines by SARS-CoV-2-infected cells. Vitamin D could exert a modulating action on the secretion of pro-inflammatory cytokines, preventing the negative consequences of the cytokine storm in terms of disease severity and/or mortality [6,7]. Vitamin D also interacts with angiotensin-converting enzyme 2 (ACE2). As ACE2 is the main entry point of SARS-CoV-2 into cells, it was postulated that vitamin D could have a role in this pathogenetic mechanism, but this is still controversial [25,26,27]. Based on these observations, many authors have investigated whether hypovitaminosis D could be linked to COVID-19. The very first results of these studies came from indirect evidence [28,29,30,31,32]. In particular, some authors found an increase in both disease severity and mortality among COVID-19 patients with hypovitaminosis D [33,34,35,36,37,38,39,40]. Very recently, some clinical trials, albeit with a limited population, showed the usefulness of high-dose vitamin D administration (cholecalciferol or calcifediol) for the treatment of COVID-19 patients [41,42]. In our study, even if our patients presented very low vitamin D serum levels, we did not observe any correlation with inflammation markers or clinical severity.

The present study has some important limitations that should be mentioned. First, the cross-sectional and retrospective design may have limited our ability to establish the causality and temporality of the associations. Second, our study population is small limiting the ability to generalize the findings to other clinical settings. A higher number of patients are needed to conclude with enough power, from subgroup analyses and to evaluate the impact of vitamin D values on mortality. Despite the small study population, post hoc power analysis revealed that the examined sample size provided adequate power for analysis of variance (90%) with a type 1 error rate less than 0.01. Third, since we have limited information on comorbidities for patients and controls, the low 25OHD levels observed may solely reflect a worse overall health status. Despite these limitations, our study has some strengths, including the consecutive enrolment of the population studied, the concomitant dosage of vitamin D levels at hospitalization, and the fact that it is the first study conducted in Sicily, which is the southern region of Italy with a high sun exposure throughout the year.

In conclusion, our retrospective study shows markedly low levels of vitamin D in patients with positive PCR for SARS-CoV-2, but it does not support a causal role of vitamin D deficiency either in infection by SARS-CoV-2 or in the clinical course of COVID-19. Other studies with a larger population and a longitudinal design are necessary to understand the real role of vitamin D in the development of COVID-19.

## Figures and Tables

**Table 1:** Clinical and laboratory characteristics of patients.

			Normal Values	SARS-CoV-2 Patients	Control Group	*p*
*n*				50	100	
Age, years				65 (24–98)	61 (22–89)	0.23
Male, *n* (%)				26 (52)	44 (44)	0.35
BMI, Kg/m^2^			18–25	27.2 (22.0–38.1)	26.4 (18.0–39.0)	0.20
Calcium, mg/dl			8.8–10.6	8.9 (6.3–9.9)	9.5 (7.7–11.0)	<0.001
25-OH vitamin D, ng/ml			≥20	12.5 (2–42)	20.5 (5–46)	<0.001
	<12 ng/ml	*n* (%)		23 (46)	15 (15)	<0.001 *
	≥12, <20 ng/ml			11 (22)	32 (32)	0.20
	≥20 ng/ml			16 (32)	53 (53)	0.015 *
PTH, pg/ml			12.0–88.0	62 (10–215)	47 (16–183)	0.03
Creatinine, mg/dl			0.51–1.29	1.03 (0.38–3.52)	0.84 (0.40–3.60)	0.52
Alkaline phosphatase, IU/l			30–120	75 (18–206)	63 (27–173) ^§^	0.37
Phosphorus, mg/dl			2.5–4.5	3.2 (1.4–5.5)	3.4 (2.1–5.0)	0.04
Albumin, g/dl			3.5–5.2	3.1 (0.1–4.2)	2.8 (2.1–4.5) ^§^	0.34
IL-6, pg/ml			0.0–6.4	12.4 (0.9–563.6)	-	
CRP, mg/l			0.0–5.0	10.2 (0.1–254.1)	-	
D-dimer, µg/l			0–250	271 (36–3599)	-	
Discharge (dead/alive)				5/45	-	
Oxygen therapy/ ventilation	None	*n* (%)		28 (56)	-	
	Nasal cannula/Venturi mask/HFNC			10 (20)	-	
	cPAP/NIV			8 (16)	-	
	Invasive ventilation			4 (8)	-	
Length of hospitalization, days				25 (7–94)	-	

Continuous variables are presented as median (range). Clinical and laboratory variables were compared using analysis of variance (ANOVA) for continuous variables and chi-squared tests for categorical variables. §: available in 50 control subjects. *: significant after Bonferroni’s correction; BMI: Body Mass Index; PTH: parathyroid hormone; IL-6: interleukin-6; CRP: C-reactive protein; HFNC: high-flow nasal cannula; cPAP: continuous positive airway pressure; NIV: non-invasive ventilation.

## Data Availability

The data presented in this study are available on request from the corresponding author.
